# On Amalgam Stoppings

**Published:** 1852-07

**Authors:** W. R. Bridgman

**Affiliations:** Norwich, England.


### ARTICLE IV.

### *On Amalgam Stoppings.* By W. R. Bridgman, Norwich, England.

That many of my professional brethren should have entirely discarded the use of all metallic compounds with mercury, for stopping teeth, I can readily understand; but, with all due deference, the propriety of such a step I am very much inclined to dispute. Not, however, that I would have it supposed, for one moment, that I am about to advocate the employment of an amalgam in any single case where gold can be effectually applied; but there are very many cases in which the latter material is quite inapplicable, and where, without having recourse to some such composition, (for I believe there is no simple nattural substance capable of answering the purpose equally well,) numbers of teeth must be either sacrificed at once, or left in a state liable to become troublesome at any future moment, and probably with the additional inconvenience of interfering with efficient mastication, but which, by a filling of amalgam carefully and judiciously applied, might stand a fair chance of becoming healthy and serviceable for many succeeding years. And as it is the duty, and ought to be the study of every one to do the utmost in his power to save a tooth, rather than to extract it, we are scarcely to be justified, I imagine, in discarding the best of our present available means, until some more efficient and less objectionable substitute has been discovered.

That there are some objections to be made against the use of mercury or quicksilver in the mouth, I am quite ready to admit, but those that are most commonly urged against it, many years experience, and close attention to the subject have led me to believe are more the results of injudicious treatment and abuse of the material, than arising from its fair and legitimate employment. One of the most obvious of its defects is the becoming converted after a short time into a *black* and *porous* mass; which, of course, so far as a stopping is concerned, renders the substance infinitely worse than useless. This, more or less, I am sorry to add, is but too commonly the state in which they are to be met with daily, and were it an insuperable fault, would at once render all further experiments useless. Such, however, is not the case. Its porosity arises mainly from an imperfection in the mode of application, and affords tolerably strong presumptive evidence that the theory of an amalgan stopping is not *generally* understood.

A consideration of the essential requisites for a stopping of any description, leads to the obvious fact that the first and most important is, that it must occupy completely the whole space of the cavity to the perfect exclusion of air and moisture, and that for a continuance of fulfilling this, it must remain unaffected by the pressure and friction of mastication, as well as unaltered by the secretions of the mouth, or by any liquid likely to be taken either with our common food or medicinally.

That gold leaf, *properly applied,* possesses these in the highest degree is indisputable, but even this substance is too often ineffectual from defective application. Decay will frequently be found to have commenced at one or more points on the side of the plug, which sufficiently proves the inaccuracy of its fit, or the insufficiency of the means adopted in the preparation of the cavity. But the former is the fault, I suspect, from the pressure being applied in the wrong direction. This, and the manner of folding and packing the gold, are scarcely of less importance than the careful preparation of the cavity itself. My own plan is to coil a strip of the leaf in such a manner as to obtain the greatest amount of soft and pliable substance in the smallest compass. To make use of a stopper not less than two-thirds the diameter of the aperture—to place the crumpled leaf across the cavity, letting it cover the sides as well as the bottom, and with a rim projecting above the edge, and to continue crossing it thus in every direction until considerable force is required to get the end of the tool inserted. This forms a "*tube*" and presses its outer surface into perfect contact with every irregularity on the sides of the cavity, and by gradually filling and well ramming its interior until it reaches nearly level with the surface of the tooth, and then gathering in the projecting rim, a plug will be formed, like a cartridge, entirely filling the interior of the cavity, and as inseparable by brushing, and ordinary wear, as if formed of a solid wire.

Now with regard to an amalgam filling, in what state are *its* component parts, and how is it generally applied?

Some kinds are in the form of small pellets, requiring to be softened by heat before they can be worked up into a paste fit for use. Others are kept in filings to be mixed with the mercury at the time of being used. The common manner of application is simply thus—the cavity having been previously prepared, and carefully dried, a lump of the paste is placed in the tooth, pressed down lightly, and wiped off with the finger. It then requires to remain some few hours undisturbed, before it becomes sufficiently hard for mastication.

If the remaining superfluous portion of the amalgam be rolled into a mass, and saved for examination when hard, its surface then will be found, under a lens of short focus, to present still the most unmistakeable evidence of its granular origin; having much the appearance of a fine grained sand stone.

But how should it be otherwise, for the mercury, unlike any common cement, has no property of itself, becoming fixed so as to fill up the interstices? The mass, when soft, may be said to be composed merely of amalgamated particles, held in mechanical suspension, and becoming hard only when it contains no more mercury than they are able to absorb.

In speaking of the alloys of mercury, Brande says: "many of these are definite and crystallizable compounds, and may be separated by gentle pressure, from the mercury in which the definite compound is suspended or dissolved." (Manual of Chemistry, page 798.) Most, if not all, these may be decomposed by being slightly heated: the mercury oozes out in the form of globules, and is then ready to perform the same part over again. Thus it is that many of the "stoppings" may be softened over and over again without injury to their setting propensity. It is to this crystallization that the porosity of the mass is owing. There is also in the act of becoming hard another change equally fatal to its effects as a useful stopping. This detrimental result, is *an increase in the bulk* of the mass, and that a very perceptible amount of expansion really does take place, may be readily proved in the following manner: Let a piece of ivory have a hole about a quarter of an inch in diameter, and rather more in depth drilled in its smooth and level surface; let this be then filled with the stopping and *immediately* scraped off level with a straight edge. When it has become hard, and, particularly, if the abominable "cadmium" has been made use of, it will be found to have risen considerably above its former level. To this defect may be attributed the falling out of a stopping so soon as it becomes hard. It is also the cause of the enamel breaking away from a tooth filled with it, as well as of the inflammation produced by the undue pressure to which a tooth often becomes subject in consequence. I think it may be fairly questioned, whether many of the supposed cases of mercurial ptyalism may not have been more the result of the latter cause, or perhaps that of a split tooth producing local irritation, than any real effects of mercurial absorption; although finely divided, and impure state offers a most favorable facility for its being acted upon by the saliva, and converted into the protoxide or perhaps some more active of its salts ; I am still unwilling to believe in its occurrence without some more satisfactory proof than I have yet met with.

After having thus noticed its leading defects, it remains to be shown how far these may be reduced to a minimum or entirely obviated in actual practice.

In the year book of facts for 1849, page 67, J. W. Buckwell's invention for preparing artificial blocks of granite is described, as consisting of the following simple process : "Fragments of a suitable stone (Portland stone for example) are guaged and sorted into sizes. These are cleaned, and carefully mixed on a board with cement in the proportion of five parts of large fragments, two of smaller ones, one of cement, and a portion of water ; but the water is in no greater quantity than will bring it to the dampness of fresh deal saw dust. This being done, the materials are put into a strong mould to the depth of about an inch-and-a-half at a time ; they are then driven together by percussion and hammered together till the water has escaped by holes pierced for that purpose in the moulds, and this process is continued till the block or pipe has attained the required magnitude. It is then taken out of the mould, and now found to be so hard as to ring when struck, and in ten days is fit for service. It will be noticed that this process is characterized by the use of fragments by the small quantity of cement employed (not one-fourth of the proportion used in common grouting) and by water, instead of fire, being made the means of bringing the fragments into close union. Mr. Faraday then noticed two scientific principles on which the success of Mr. Buckwell's process greatly depends.

1st. *The use of water in effecting the approximation of the particles, and the exclusion of the air.*

2nd. *The effect of percussion in bringing particles together.*

Mr. Faraday noticed, that simple pressure will not displace interstitial air, but that a blow will.

Here we have in the preceding process, a result obtained perfectly analogous to the disideratum in a mercurial stopping, the greatest amount of density produced with the smallest quantity of cementing material; and, although it is neither possible, nor requisite, to use any considerable amount of "percussion" in stopping a tooth, yet, a kind of gentle jerk may be given almost imperceptibly, which will have the same effect. If a portion of stopping scarcely soft enough to adhere by pressure between the finger and thumb, be placed in a cavity, and treated thus, it will soon become "pulpy" on the surface, with a substratum of the filings, or solid part, jammed into a hard and unyielding mass beneath it. The mercury performing the same office, as the water in the former case. If this outer creasing substance be taken off, and either pressed dry, or rendered stiff with additional filings, and again applied until the cavity be filled, there will be found to have been but a very small quantity of the mercury retained in the stopping. Too little, perhaps, it may be thought, to render the mass sufficiently binding; but there need be no apprehension of this when *clean* filings of most of the ductile metals may be made to cohere by simple pressure alone. An instance of this, is the "sponge" gold, but the ephemeral fame of which, as a preferable form of the metal for stopping, is fast and most deservedly on the wane.

To get rid of the air bubbles, which are apt to be confined between the stopping and the tooth, particularly in the angles of the cavity, it is necessary to coat the latter first, with a very thin layer of the cement, worked about with a small tool and *sliding* instrument in every direction. After this, it may be filled up in the above manner with every chance of success.

If the piece of ivory containing the amalgam filling, used in the former experiment, be now placed within a clear glass jar or cup, with the stopping uppermost, and covered to the extent of about half an inch with cold boiled water, and then placed under the receiver of an air pump; on the air being withdrawn slowly, bubbles of air will be seen to emerge from the edge of the stopping, between it and tbe bone, and which, in the mouth, would soon be replaced with saliva.* Let a plug of gold, prepared in the most careful manner, be now substituted for the preceding, and it will be seen how extremuly difficult it is to form an air tight stopping with this material *when used dry*, in comparison with the same *previously dipped in water*, and allowing this to take the place of air within its folds. The compressibility of the air, and its expansion on the removal of the pressure, renders the mass frothy, while the *non*-compressibility of the water, causes it to be squeezed out, and the plug to become far more dense than by any other means. I would strongly urge upon all, the desirability of well testing in this manner the merits of every preparation or material, and be so used before trusting it in the mouth. Had such precaution been taken with the "cadmium," and which I fortunately did on its first introduction, it would have prevented much discredit to the profession, generally, as well as loss of fame to many of its professors individually.

### ARTICLE V.

### *Dental Inventions and Pretensions.*

* To avoid the possibility of a portion of the air coming from within the substance of the matrix, as well as from the stopping, it would be necessary to use *glass cells*, (pieces of barometer tube closed at one end would do,) but for a rough experiment, the above answers every purpose.

